# RapaCaspase-9-based suicide gene applied to the safety of IL-1RAP CAR-T cells

**DOI:** 10.1038/s41434-023-00404-2

**Published:** 2023-05-12

**Authors:** Lucie Bouquet, Elodie Bôle-Richard, Walid Warda, Mathieu Neto Da Rocha, Rim Trad, Clémentine Nicod, Rafik Haderbache, Delphine Genin, Christophe Ferrand, Marina Deschamps

**Affiliations:** 1 Univ. Bourgogne Franche-Comté, INSERM, EFS BFC, UMR1098, RIGHT Interactions Greffon-Hôte-Tumeur/Ingénierie Cellulaire et Génique, F-25 000 Besançon, France; 2 CanCell Therapeutics, 25 000 Besançon, France

**Keywords:** Immunotherapy, Haematological cancer

## Abstract

Even if adoptive cell transfer (ACT) has already shown great clinical efficiency in different types of disease, such as cancer, some adverse events consistently occur, and suicide genes are an interesting system to manage these events. Our team developed a new medical drug candidate, a chimeric antigen receptor (CAR) targeting interleukin-1 receptor accessory protein (IL-1RAP), which needs to be evaluated in clinical trials with a clinically applicable suicide gene system. To prevent side effects and ensure the safety of our candidate, we devised two constructs carrying an inducible suicide gene, RapaCasp9-G or RapaCasp9-A, containing a single-nucleotide polymorphism (rs1052576) affecting the efficiency of endogenous caspase 9. These suicide genes are activated by rapamycin and based on the fusion of human caspase 9 with a modified human FK-binding protein, allowing conditional dimerization. RapaCasp9-G- and RapaCasp9-A-expressing gene-modified T cells (GMTCs) were produced from healthy donors (HDs) and acute myeloid leukemia (AML) donors. The RapaCasp9-G suicide gene demonstrated better efficiency, and we showed its in vitro functionality in different clinically relevant culture conditions. Moreover, as rapamycin is not pharmacologically inert, we also demonstrated its safe use as part of our therapy.

## Introduction

Adoptive cell transfer (ACT) is an interesting therapy for cancer management. Antigen-specific T cells present as tumor-infiltrating lymphocytes have potential uses in ACT, but T cells can also be genetically engineered for ACT, such as transgenic T cell receptor (TCR)- and chimeric antigen receptor (CAR)-T cells [1]. Nevertheless, some adverse immunological phenomena can occur with this type of therapy, such as cytokine release syndrome (CRS), neurotoxicity and on-target/off-tumor or off-target/off-tumor toxicities [2–8]. To abrogate and limit adverse effects, the establishment of a suicide gene is needed as it was well described for managing neurotoxicities in CD19 CART immunotherapy clinical trial [9].

Several suicide gene systems, or safety switches, have been developed to improve the safety of ACT by allowing quick elimination of engineered cells if needed. The perfect suicide gene activation system would be based on an activating agent that is biologically inert, bioavailable and characterized by acceptable or limited toxicity. Some safety switches target surface molecules, such as the huEGFRt/cetuximab and CD20/rituximab systems: huEGFRt is a truncated receptor tyrosine kinase that is not expressed by hematopoietic or lymphopoietic cells and is targeted by cetuximab, a specific monoclonal antibody. CD20 is targeted by rituximab and allows efficient depletion of gene-modified T cells (GMTCs) but not healthy cells. Moreover, therapeutic monoclonal antibodies are required to be present in sufficient concentrations to be efficient, and these levels may not be reached in all tissue distributions [10–12]. A suicide gene can also use a transgenic enzyme to activate a prodrug system, such as cytosine deaminase or herpes simplex virus thymidine kinase (HSV-TK). Cytosine deaminase activates 5-fluorocytosine, inducing 5-fluorocytosine conversion into 5-fluorouracil, which is then transformed into potent pyrimidine antimetabolites. This system induces cell proliferation inhibition and cell death [10, 11]. HSV-TK activates ganciclovir and is used as a suicide gene after hematopoietic stem cell transplantation. Ganciclovir is converted into a monophosphorylated molecule and then metabolized into toxic triphosphate, inhibiting DNA synthesis in dividing cells and ultimately inducing cell death. This system has been tested in the clinic but is limited by the use of ganciclovir in cases of cytomegalovirus infection [10, 13–15]. Sometimes, the HSV-TK-directed immune response leads to HSV-TK-transduced cells elimination, which compromises the persistence of infused T cells [16]. A suicide gene system involving inducible Caspase 9 (iCaspase9) has also been tested in the clinic, and its utility has widely been proven [17]. iCaspase9 is a fusion of a mutated 12-kDa FK506 binding protein (FKBP12) with the catalytic domain of caspase 9. FKBP12 is mutated to allow for the docking of AP1903, which is a small-molecule chemical inducer of dimerization (CID) [8, 10, 16]. CID administration induces cross-linking and activation of proapoptotic target molecules, leading to cell apoptosis. Unfortunately, AP1903 is not a licensed pharmaceutical agent and cannot be used for therapy. To create a system with a widely available immunosuppressive pharmaceutical to induce a suicide gene, Stavrou’s team [12] developed and optimized a rapamycin-activated caspase 9 (RapaCaspase9) gene, which is induced by rapamycin as the CID. Rapamycin induces heterodimerization of FKBP12 with rapamycin fragment binding domain (FRB) or mammalian target of rapamycin, allowing activation of the fused catalytic domain of caspase 9. Even if rapamycin is not pharmacologically inert, its use is completely different from its standard use, and it will be a great alternative to improve the safety of T-cell therapy.

A few years ago, our team developed a new medical drug candidate, a CAR targeting the cell-surface IL-1RAP protein, which has proven efficiency in acute AML [18] and chronic myeloid leukemia [19] in vitro and in vivo with mice model but has yet to be evaluated in a clinical trial. Nevertheless, this therapy lacks a clinically applicable suicide gene system, and RapaCaspase9 could be a great alternative. As a single-nucleotide polymorphism (SNP) Ex5+32G>A (rs1052576) showed a functional impact on endogenous Caspase 9, with a protective effect for the AA alleles in different cancers [20–22] and negative impacts for the GG allele [23], we assessed two RapaCaspase9 suicide gene systems to improve the safety of our IL-1RAP CAR-T cells.

## Materials and methods

### Cell culture

AML samples were collected from patients with AML at follow-up or diagnosis included, after consent, in the approved AML samples collection, CAR-LAM, N° CPP: CPP2019-03-022a, France). Peripheral blood mononuclear cells (PBMCs) were collected from HDs at the French Blood Center (Besançon, France) as apheresis kit preparations after obtaining written informed consent and following internal guidelines. PBMCs were isolated by centrifugation on a Ficoll gradient. T cells from healthy donor buffy coats (*n* = 3) were used for CAR-T-cell production. Donors from the French Blood Establishment provided written informed consent, and the study was conducted in accordance with appropriate ethical guidelines (Declaration of Helsinki) and approved by the local ethics CPP-Est committee (France).

### Lentiviral and retroviral plasmid constructs

Retroviral iCaspase-9-G (iCasp9-Gr) and iCaspase-9-A (iCasp9-Ar) constructs were generated by ligation of the insert of interest with a vector (pSFG, containing a Moloney murine leukemia virus (MMLV) –derived 5 0 -LTR and a chimeric 3 0 -LTR that contains the myeloproliferative sarcoma virus (MPSV) 27 promoter/enhancer). Lentiviral RapaCaspase-9-G (RapaCasp9-G) and iCaspase-9 (iCasp9-G) constructs (pSDY, a self-inactivating lentiviral construct containing EF1a promoter) were generated as previously described [19]. The RapaCaspase-9-A (RapaCasp9-A) construct was obtained by site-directed mutagenesis of the RapaCaspase-9-G construct with QuikChange II XL Site-Directed Mutagenesis (Agilent, Santa Clara, California, USA) according to the manufacturer’s instructions. To ensure that the directed mutagenesis was correctly carried out, PCR targeting the suicide gene was performed, and the amplification products underwent restriction fragment length polymorphism by the BstUI enzyme (restriction site CG|CG). Sanger sequencing was also performed to validate the site-directed mutagenesis with a BigDye® terminator v3.1 cycle sequencing kit (ThermoFisher, Waltham, Massachusetts, USA) according to the manufacturer’s instructions. After purification with a NucleoSEQ kit (Macherey-Nagel, Hoerdt, France), samples were run on a 3500 Genetic Analyzer (ThermoFisher) and analyzed using Sequencing Analysis software version 5.2 (Applied Biosystems, Waltham, Massachussets, USA). Briefly, the vectors carried a third-generation CAR targeting the IL-1RAP protein, a gene encoding surface-expressed truncated human CD19 (∆CD19, 939pb, 313 AA) (for RapaCasp9-G, RapaCasp9-A, iCasp9-G and iCasp9-Gr) or truncated human wild type CD34 (∆CD34, 981pb, 327 AA) (iCasp9-Ar) for monitoring transduction efficiency and the suicide gene RapaCasp9-G, RapaCasp9-A, retroviral iCasp9-G or retroviral iCasp9-Ar suicide genes induction. A schematic representation of the different vectors is provided in Supplementary Fig. S1.

### Production of retroviral and lentiviral construct supernatants and transduced T cells

Retroviral supernatants were harvested at 48 h and 72 h from subconfluent tritransfected HEK-293T cells (pGP, pE-Eco and transgene plasmids) and used to transduce PG13 cells. CD34^+^ and CD19^+^ PG13 cells were sorted by flow cytometry. Retroviral supernatants were then harvested at 48, 72 and 96 h from subconfluent PG13 cells. Lentiviral supernatants were harvested at 48 h and 72 h from subconfluent tritransfected HEK-293T cells (pMDG, psPAX2, and transgene plasmids).

### Production of lentivirally or retrovirally transduced T cells

T cells from HDs or AML sample patients were activated and selected in two different ways. The first process included activation and selection with CTS™ Dynabeads™ CD3/CD28™ (ThermoFisher) according to the manufacturer’s instructions. After 2 days of expansion in RPMI-1640 medium supplemented with 8% human serum and 500 UI/mL IL-2, T cells were transduced with a retroviral or lentiviral supernatant. For the second process, T cells were transduced with a lentiviral supernatant after CD4^+^/CD8^+^ selection by magnetic beads (Miltenyi Biotec, Bergish Gladbach, Germany) and CD3/CD28 activation by T Cell TransAct^TM^ (Miltenyi Biotech), according to the manufacturer’s instructions, following a 1-day expansion period. Cells were cultured in TexMACS Good Manufacturing Process (GMP) Medium supplemented with IL-7 and IL-15 and used for CliniMACS Prodigy® (Miltenyi Biotech) [24]*.* Transduction efficiency was evaluated after 7 days by flow cytometry, and cells were expanded for 9 days. Control cells from the same donor or patient were prepared following the same protocol.

### Rapamycin/AP1903-induced killing

Transduced cells were seeded at a density of 1 × 10^5^ to 2 × 10^5^ cells in 96-well plates. RapaCasp9-G-expressing GMTCs were stimulated or not with plate-bound [25] IL-1RAP for 24 h before suicide gene activation, as described below. For treated (TT) cells, cells were exposed to rapamycin at concentrations between 0.1 and 109 nM (Sigma‒Aldrich, Saint-Louis, Missouri, USA; CliniSciences, Nanterre, France) for the RapaCaspase9 suicide genes and to AP1903 at 10 nM (Bellicum Pharmaceuticals, Houston, Texas, USA) for the iCaspase9 suicide genes. Untreated (UT) cells were cultured with medium containing 1% DMSO (RapaCasp9) or in the absence of drug (iCasp9). Cells were exposed for 0–72 h at 37 °C. Cell death was evaluated by Annexin-V-FITC, 7-aminoactinomycin D (7-AAD), anti-CD3-VioBlue and anti-CD19-APC or anti-CD34-APC labeling for flow cytometry: the percentage of killed cells was calculated upon normalization to the percentage of live CD3^+^/CD19^+^ cells in the respective untreated control. Acquisition for 90 s with standard flow cytometry tubes or of 5000–10,000 events with TruCount tubes (BD BioSciences, Le Pont-de-Claix, France) was performed.

### Flow cytometry

The T-cell transduction efficiency for HDs or AML patients was determined by labeling using anti-CD3-VioBlue (Miltenyi Biotec) and anti-CD19-APC (Sony Biotechnology, San José, California, USA) or anti-CD34-APC (Sony Biotechnology). The expression of the IL-1RAP CAR was assessed by labeling with a biotinylated IL-1RAP protein (Diaclone, Besançon, France), followed by labeling with Streptavidin-PE (Miltenyi Biotec). A proliferation assay was performed with eFluor V450 (ThermoFisher) and anti-CD3-VioBlue (Miltenyi Biotech) labeling. A CD107a degranulation assay was performed by labeling cells with anti-CD107a-PE (BD BioSciences), anti-CD3-VioBlue, anti-CD19-APC and anti-CD8-FITC (Diaclone). Labeled cells were collected by an LSR Fortessa flow cytometer (BD Biosciences) and analyzed using FACSDIVA software (BD Biosciences).

### Impact on insensitive T cells: T-cell proliferation assay and impact on transgene expression

To assess the impact of rapamycin on insensitive T cells, untransduced cells were labeled with eFluor v450 or left unlabeled according to the manufacturer’s instructions and transferred to 24-well plates at a density of 1 × 10^5^ cells with or without rapamycin (109 nM). Unlabeled and labeled cells were weaned off rapamycin 48 h after treatment began based on the rapamycin half-life [26] and proliferation was assessed by flow cytometry 96 h after treatment began for the labeled cells. The unlabeled cells were labeled with eFluor V450 48 h after weaning, and proliferation was assessed 96 h later.

Untransduced cells were also treated with rapamycin for 48 h and then weaned off rapamycin. Proliferation was assessed by Trypan Blue counting every 24 h for 168 h.

To assess the impact of rapamycin on transgene expression, rapamycin-insensitive cells were exposed to 109 nM rapamycin for 24 h. The medium culture was weaned off rapamycin, and CD19 and CAR expression were then evaluated immediately after weaning, 72 h and 120 h after weaning.

### Statistical analysis

All data are presented as the average ± standard deviation (SD) on graphs. Unpaired Student *t* test and two-way Anova were used to determine the statistical significance of differences between samples, and a *P*-value less than 0.05 was accepted as a statistically significant difference. Data were analyzed and plotted using GraphPad Prism.

## Results

### The iCasp9-G construct showed a correct suicide gene efficiency

**Fig. 1:**
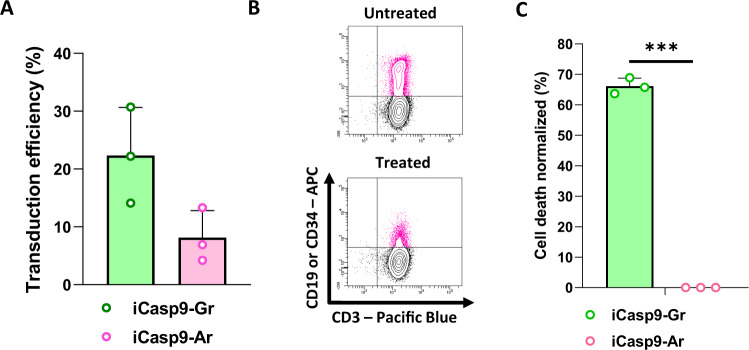
Generation of iCasp9-Gr- and iCasp9-Ar-expressing GMTCs and suicide gene functionality. **A** Retroviral transduction efficiency of T cells determined by flow cytometry. T cells from HDs were genetically modified. Transduction efficiency was analyzed on day 7 after the beginning of production. Mean ± SD of three independent experiments. **B** Representative dot plots for AP1903-induced killing determined by flow cytometry. **C** Cell death percentages of cells expressing iCasp9-G measured 24 h after 10 nM AP1903 exposure. Normalized to control cells (untreated cells). Cell death was evaluated by Annexin-V/7-AAD labeling and gating on CD3^+^/CD19^+^ cells (−Gr) or CD3^+^/CD34^+^ cells (−Ar) by flow cytometry. Mean ± SD of three independent experiments. ****P < 0.0001 with unpaired Student *t* test.

The two retroviral iCaspase9 plasmids were used to produce iCasp9-Gr- and iCasp9-Ar-expressing GMTCs. To confirm the correct constructs were created, we performed PCR targeting the suicide gene RapaCaspase9 and then restriction length fragment polymorphism with the BstUI enzyme (Supplementary Fig. S2A), which recognized the restriction site CG|CG and allowed the identification of the plasmid SNP. RapaCasp9-G presented as digested bands at 287 and 246 base pairs (bp), unlike RapaCasp9-A, which presented as only an undigested band at 533 bp, validating the site-restricted mutagenesis process. The results were confirmed by the SNaPshot technique, and lentiviral supernatants were produced to transduce T cells (Supplementary Fig. S2B and C, respectively). We were able to transduce primary T cells with iCasp9-Gr and iCasp9-Ar retroviral supernatants, as shown in Fig. 1A. AP1903-induced killing of transduced cells (CD3^+^/CD19^+^ for iCasp9-Gr cells; CD3^+^/CD34^+^ for iCasp9-Ar cells) from three different HDs donors was evaluated by flow cytometry. Representative flow cytometry dot plots are shown Fig. 1B and the data are shown in Fig. 1C. We observed dysfunctionality of the iCasp9-Ar suicide gene with no AP1903-induced killing in comparison with the iCasp9-Gr suicide gene, showing an impact of the SNP on suicide gene induction.

### The SNP G/A has no significant impact on RapaCaspase-9 suicide gene efficiency with a lentiviral vector

**Fig. 2:**
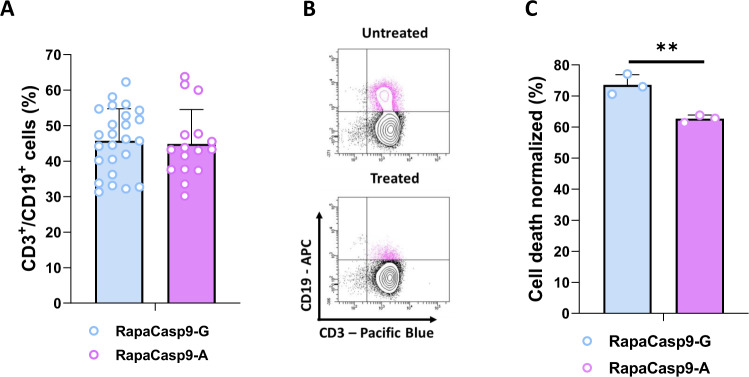
Generation of functional RapaCasp9-G– or RapaCasp9-A–expressing GMTCs. **A** Lentiviral transduction efficiency of T cells determined by flow cytometry. T cells from HDs were genetically modified. Transduction efficiency was analyzed on day 7 after the beginning of production. Mean ± SD of twenty-six (−G) and sixteen (−A) independent experiments. **B** Representative dot plots of rapamycin-induced killing determined by flow cytometry. **C** Cell death percentages of cells expressing RapaCasp9-G 24 h after 1 nM rapamycin exposure. Normalized to control cells (DMSO exposure). Cell death was evaluated by Annexin-V/7-AAD labeling and gating on CD3^+^/CD19^+^ cells by flow cytometry. Mean ± SD of three independent experiments. **P < 0.01 with unpaired Student *t* test.

We validated lentiviral constructs following the procedure described for retroviral constructs after site-directed mutagenesis by targeting the SNP Ex5+32G (rs1052576), changed the nucleotide G to A in the RapaCasp9-G plasmid (Supplementary Fig. S3A, B) and produced lentiviral supernatant (Supplementary Fig. S3C). Using our lentiviral constructs (Supplementary Fig. S1B), we were able to transduce T cells from HDs with RapaCasp9-G or RapaCasp9-A supernatants (Fig. 2A). To validate our constructs, we assessed the functionality of the RapaCaspase9 suicide genes by exposing transduced cells to rapamycin at a minimal effective dose [12] for 24 h. Representative flow cytometry dot plots are shown Fig. 2B. Rapamycin-induced killing was evaluated by flow cytometric analysis of transduced cells (CD3^+^/CD19^+^) from three different PBMC donors, and the data are shown in Fig. 2C. We observed better rapamycin-induced killing for the RapaCasp9–G construct than for the RapaCasp9–A construct, suggesting better suicide gene induction for the –G system.

### The RapaCasp9-G construct is more efficient than the RapaCasp9-A construct

**Fig. 3:**
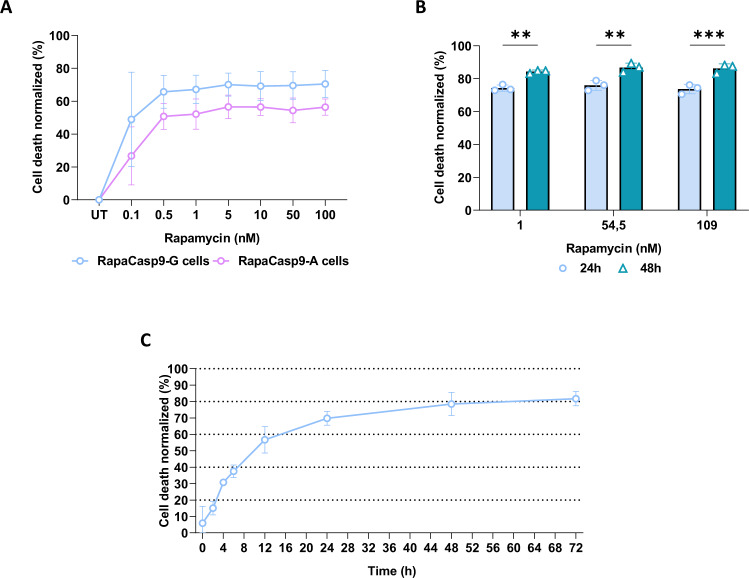
Functional dose improvement for suicide gene induction in RapaCasp9-expressing GMTCs. **A** Killing curve after 24 h of exposure to rapamycin at doses ranging from 0.1 nM to 100 nM. Mean ± SD of three independent experiments. **B** Cell death percentages of RapaCasp9-G-expressing GMTCs 24 and 48 h after rapamycin exposure. Cells were exposed to rapamycin at a published dose (1 nM) or clinical doses equivalents (54.5 and 109 nM). Normalized to control cells (DMSO exposure). Cell death was evaluated by Annexin-V/7-AAD labeling and gating on CD3^+^/CD19^+^ cells by flow cytometry. Mean ± SD of three independent experiments. **P < 0.01; ***P < 0.001 with Two-way Anova test. **C** Cell death percentages of RapaCasp9-G-expressing GMTCs during 72 h after rapamycin exposure. Cells were exposed to 109 nM rapamycin and normalized to control cells (DMSO treated). Cell death was evaluated by Annexin-V/7-AAD labeling and gating on CD3^+^/CD19^+^ cells by flow cytometry. Mean ± SD of three independent experiments.

To determine the best RapaCaspase9 suicide gene to improve the safety of our therapy, RapaCasp9-G- and RapaCasp9-A-expressing GMTCs were exposed to increasing rapamycin doses (0.1–100 nM) for 24 h (Fig. 3A). The cells reached a plateau of rapamycin-induced killing between 1 and 5 nM, with greater cell death rates for the RapaCasp9 than RapaCasp9-A construct: these results showed the greater efficiency of the –G construction for all evaluated dilutions.

In the context of suicide gene use after the onset of undesirable side effects of cell therapy, rapamycin pills, in the form of tablets, will be taken. To evaluate these rapamycin doses, we tested 54.5 nM and 109 nM in comparison with the minimal effective dose for induction.

The relationship between rapamycin tablets and the molarity is respectively 0.25 mg (take of half a tablet) for 54.5 nM and 0.5 mg (whole tablet) for 109 nM. Calculation was extrapolated assuming that there are 5 liters of blood volume in an adult human. Because of the better suicide gene efficiency and to project ourselves into the context of a clinical trial, only RapaCasp9-G-expressing GMTCs were treated with these doses, and the cell death percentage was evaluated 24 and 48 h after rapamycin exposure for three different HDs donors (Fig. 3B). The data showed a significant increase in the cell death percentage between 24 h and 48 h after exposure, regardless of the dose, validating the use of clinical doses and notably the 109 nM dose for the rest of the study. To ensure of the same efficiency of induction between rapamycin pills (Rapamune®, Pfizer), which will be used in conditions of clinical trial, with rapamycin supplied for research use, pills were crushed and used to treat RapaCasp9-G-expressing GMTCs in comparison with reconstituted powder rapamycin. We showed that cell death percentages of RapaCasp9-G-expressing GMTCs confirmed the same efficiency of induction with both inducers (Supplementary Fig. S4).

To evaluate the induction speed of the RapaCasp9-G suicide gene, RapaCasp9-G-expressing GMTC cell death was evaluated at 0, 2, 4, 6, 12, 24, 48 and 72 h after rapamycin exposure. As shown in Fig. 3C, RapaCasp9-G-expressing GMTCs reached a plateau at 48 h after exposure, but a high cell death percentage was already observed at 24 h after rapamycin exposure, showing the great speed of suicide gene induction.

### Rapamycin induces a temporary slowdown in proliferation and transitory transgene expression changes

**Fig. 4:**
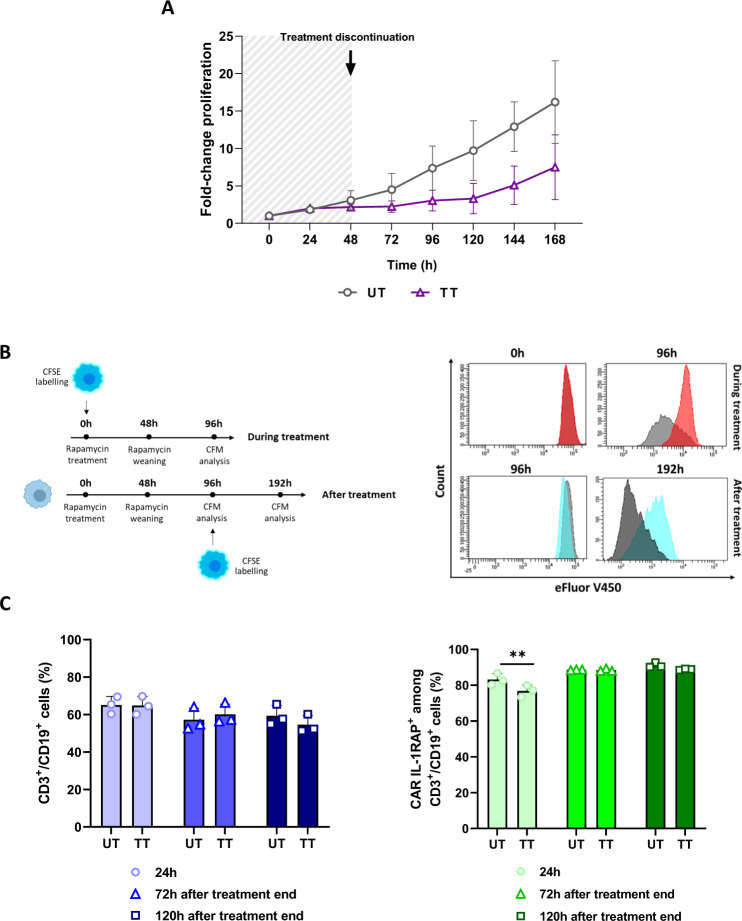
Rapamycin effects on different insensitive cells. **A** Fold-change in the proliferation of untransduced cells treated with 109 nM rapamycin (TT) or not (UT) and weaned after 48 h measured by Trypan blue counting. Counting was performed each day for 168 h. Mean ± SD of three independent experiments. **B** Left, experimental design for the proliferation analysis by carboxyfluorescein succinimidyl ester (CFSE) labeling during and after rapamycin treatment; right, representative histograms of CFSE labeling of T cells treated with rapamycin and weaned after 48 h. CD3^+^/CFSE^+^ cells were analyzed by flow cytometry 96 h after rapamycin treatment and 192 h after weaning. **C** The impact of rapamycin on CD19 (left) and CAR IL-1RAP (right) expression in iCasp9-expressing genetically modified T cells. Cells were treated for 24 h with 109 nM rapamycin (TT, treated) or not (UT, untreated) and then weaned. CD3^+^/CD19^+^/CAR IL-1RAP^+^ cells were analyzed 24 h after treatment began and 72 h and 96 h after weaning. **P < 0.01; with Two-way Anova test.

To evaluate the impact of rapamycin on insensitive cell proliferation, untransduced cells were treated for 48 h and then weaned. Proliferation was assessed by Trypan Blue counting for 168 h. For rapamycin-exposed cells, a temporary slowdown in proliferation was observed 120 h after rapamycin exposure, and then proliferation resumed (Fig. 4A). To confirm the obtained results, eFluor V450-labeled untransduced cells were exposed to rapamycin and then weaned after 48 h of exposure (Fig. 4B); these cells showed only a temporary slowdown in proliferation, confirming previous results.

To evaluate the impact of rapamycin on transgene expression, rapamycin-insensitive cells were exposed to rapamycin for 24 h and then weaned (Fig. 4C). CD19 and CAR expression were then evaluated after exposure and after weaning. No significant difference was observed in the CD3^+^/CD19^+^ cell percentages between untreated (UT) and treated (TT) cells at any time. However, a temporary significant difference in the CD3^+^/CD19^+^/CAR IL-1RAP^+^ cell percentages between TT and UT cells was observed only 24 h after rapamycin exposure; indeed, 72 and 120 h after weaning, similar percentages were observed between the two conditions. Even if rapamycin induces transitory transgene expression changes, it does not impact suicide gene induction in sensitive cells and induces only a temporary slowdown.

### Initial IL-2 or IL-7/IL-15 activation do not impact RapaCasp9-G-expressing GMTC suicide gene induction in HDs and AML patients, as well as the freezing process

As the production process for CAR-T cells targeting IL-1RAP must be GMP compliant, we evaluated the impacts the activation process and of freezing on suicide gene induction.

**Fig. 5:**
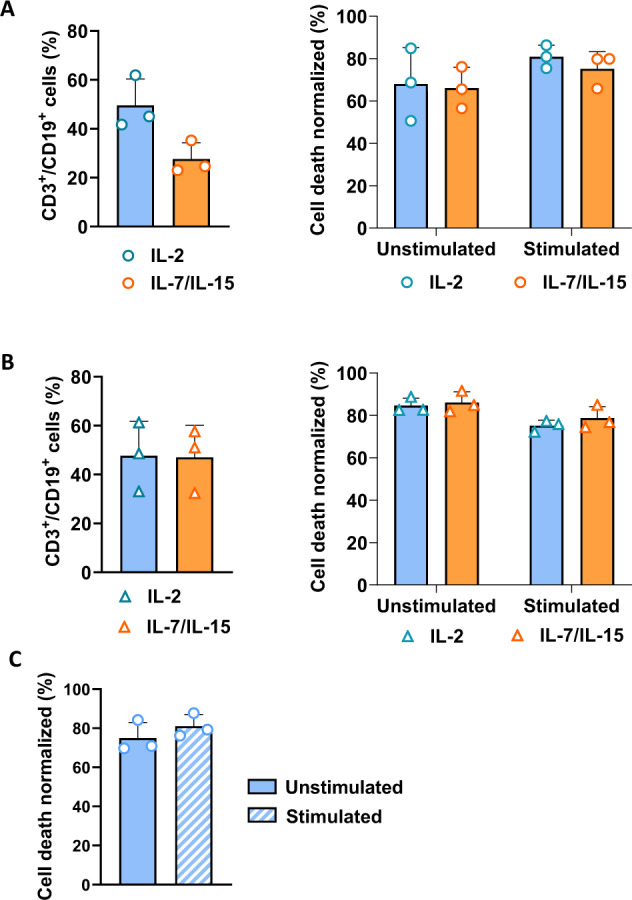
Functional validation of the RapaCasp9-G suicide gene in RapaCasp9-G-expressing GMTCs from HDs and AML patients in clinically relevant conditions. **A** Left, lentiviral transduction efficiency of T cells in IL-2 and IL-7/IL-15 conditions determined by flow cytometry. T cells from HDs were genetically modified. Transduction efficiency was analyzed on day 7 after the beginning of production. Mean ± SD of three independent experiments; right, cell death percentages of RapaCasp9-G-expressing GMTCs stimulated with plate-bound IL-1RAP or left unstimulated for 24 h; the percentages were measured 24 h after rapamycin exposure. T cells were cultured in RPMI medium supplemented with IL-2 or in TexMacs medium supplemented with IL-7/IL-15. Normalized to control cells (DMSO exposure). Mean ± SD of three independent experiments. **B** Left, lentiviral transduction efficiency of T cells in IL-2 or IL-7/IL-15 conditions determined by flow cytometry. T cells from AML patients were genetically modified. Transduction efficiency was analyzed on day 7 after the beginning of production. Mean ± SD of three independent experiments; right, cell death percentages of RapaCasp9-G-expressing cells stimulated with plate-bound IL-1RAP or left unstimulated for 24 h prior to culture in IL-2 or IL-7/IL-15 conditions; the percentages were measured 24 h after rapamycin exposure. Normalization to control cells (DMSO exposure). Mean ± SD of three independent experiments. **C** Cell death percentages of freshly thawed RapaCasp9-G-expressing GMTCs stimulated with plate-bound IL-1RAP-coated or left unstimulated for 24 h; the percentages were measured 24 h after rapamycin exposure. Normalized to control cells (DMSO exposure). Mean ± SD of three independent experiments.

To evaluate suicide gene induction in cells produced in clinically relevant conditions, RapaCasp9-G-expressing GMTCs were produced in research conditions with CD3^+^/CD28^+^ activation/sorting in IL-2-supplemented medium (IL-2) or in clinically relevant conditions with CD4^+^/CD8^+^ sorting and TransAct^TM^ activation in IL-7/IL-15-supplemented medium (IL-7/IL-15) (Fig. 5A top). RapaCasp9-G-expressing GMTCs were stimulated for 24 h with plate-bound IL-1RAP and exposed to rapamycin. The cell death percentage of CD3^+^/CD19^+^ cells was evaluated 24 h later. Higher but not significantly different cell death percentages were observed between stimulated and unstimulated conditions, and there was a nonsignificant difference between the two culture conditions (Fig. 5A bottom), indicating the preservation of functionality with different activation processes, notably the clinically relevant conditions. To assess the use of a suicide gene and evaluate activation process impact in cells from leukemic donors, RapaCasp9-G-expressing GMTCs were also produced from AML patients with a sufficient transduction efficiency in both IL-2 and IL-7/-15 conditions (Fig. 5B top). RapaCasp9-G-expressing GMTCs were stimulated with plate-bound IL-1RAP, as confirmed by a CD107a degranulation assay (Supplementary Fig. S5A). RapaCasp9-G-expressing GMTCs were exposed to rapamycin for 24 h after IL-1RAP stimulation (Fig. 5B bottom). Rapamycin-induced killing of transduced cells (CD3^+^/CD19^+^) was evaluated for three different AML donors for IL-2 and IL-7/IL-15 conditions. Great cell death percentages were reached in both conditions, and there was not a significant difference between the unstimulated and stimulated conditions of IL-2 and IL-7/IL-15 conditions. These results confirmed the suicide gene functionality of GMTCs from AML patients and showed that there is no impact of the production process on suicide gene functionality.

Because, for clinical trial, the drug product of IL-1RAP CART-cells will be the cryopreserved drug substance, we evaluate the impact of freezing process on suicide gene induction. RapaCasp9-G-expressing GMTCs produced from HDs were frozen on day 9 after activation and then thawed to perform functional tests. After thawing, the modified cells were directly stimulated or not with a plate-bound IL-1RAP protein, as confirmed by a CD107a degranulation assay (Supplementary Fig. S5B). The RapaCasp9-G-expressing GMTCs were then exposed to rapamycin after IL-1RAP stimulation, and rapamycin-induced killing was evaluated for transduced cells (CD3^+^/CD19^+^) from three different HDs. Even after thawing, RapaCasp9-G-expressing GMTCs reached high cell death percentages, with a better cell death percentage for the stimulated condition than for the unstimulated condition (Fig. 5C), confirming the functionality of the suicide gene despite the freezing/thawing process.

## Discussion

The safety of ACT is very important because of the potential for on-target/off-tumor, off-target and CRS toxicities; indeed, the on-target/off-tumor toxicities caused by CAR-T cells directed against HER2 [27] or a transgenic TCR directed against MAGE-13 protein led to respiratory failure and neurotoxicity [28]. Moreover, off-target toxicities, such as nonspecific recognition by the TCR, lead to off-target/off-tumor toxicities, such as a MAGE-A3-specific transgenic TCR recognizing the Titin protein and causing cardiac death [29, 30]. Other toxicities such as CRS, which induces more or less dangerous symptoms, and the neurotoxicities notably reported with CD19-specific CAR-T cells [31] have to be managed, and suicide genes are a supplementary safety measure in certain cases.

Our CAR-T-cell targeting the cell-surface protein IL-1RAP was functionally validated in chronic myeloid leukemia and AML and will be evaluated in a clinical trial; and its suicide gene functionality, that of the iCaspase9 suicide gene, was already validated. However, the inducer of iCaspase9 suicide gene is not yet available as an approved drug which limits the use of this suicide gene system in the clinic. To make the use of the suicide gene in the clinic easier, the RapaCaspase9 suicide gene would be a great alternative: it is induced by a well-known and already use drug (rapamycin), available as off-the-shelf pharmaceutical licensed for commercial sale worldwide with a good biodistribution. Moreover, it is known to have an anti-cancer activity, even in the case of activation of the safety switch, the dose will be very low. This medication was approved in 1999 for prevention of renal graft rejection [32] and exerts immunosuppressive activity: this activity could create a real interest because of the high T-cell activity in ACT but will not be harmful given the needed dose for suicide gene activation in comparison to that for prevention of renal graft rejection [12].

The RapaCaspase9 suicide gene is a fusion of a mutated FKBP12 protein with the rapamycin FRB of mammalian target rapamycin. FKBP12-FRB is fused to the catalytic domain of Caspase 9, with a short linker between the FRB and FKBP12. Physiologically, caspase 9 is activated by oligodimerization of its caspase activation and recruitment domain with the apoptosome. For the RapaCaspase9 suicide gene, rapamycin induces heterodimerization of FRB and FKBP12, allowing the activation of the caspase 9 catalytic domain.

Caspase 9 plays an important role from the beginning to the end of an individual’s life; indeed, it has been demonstrated that decreased apoptosis, caused by mutations in the caspase 9 gene, results in embryonic lethality and defective brain development [20, 33–35]. The importance caspase 9 was also confirmed by the existence of different SNPs in endogenous caspase 9 that are described to have functional impacts on its efficiency and important consequences. This is the case for the SNP Ex5+32G>A (rs1052576), which shows a protective effect for the AA alleles in different cancers, such as glioma brain tumors [20], non-small cell lung cancer [21], and B-cell lymphoma [22], and a negative impact for the GG allele indicated by a survival reduction [23].

In this study, the SNP Ex5+32G>A (rs1052576) was incorporated into the iCaspase9 and RapaCaspase9 suicide gene improving the safety of IL-1RAP-targeting CAR constructs to assess its impact and optimize the efficiency of the suicide gene.

Our first works on suicide genes were developed with retroviral constructs containing the iCaspase9 suicide gene with one or the other SNP: the SNPs showed an important impact on suicide gene efficiency, but unlike endogenous caspase 9, the G nucleotide is responsible for better induction of cell death. With biotechnological advances, retroviral constructs, allowing bad and more difficult transductions, are progressively being replaced by lentiviral constructs; indeed, the ability of retroviral particles to transduce only cells undergoing division considerably reduces the efficiency of primary T-cell genetic modification. In the context of improving the safety of our CAR-T cells, RapaCaspase9 suicide genes with the SNP Ex5+32G>A (rs1052576) were introduced into lentiviral constructs: unlike for the retroviral constructs, the SNP did not show an impact on induced cell death with RapaCaspase9, but the –G construct presented a better efficiency with the minimal effective dose determined previously [12]. Similar results were also observed with a range of doses of rapamycin, confirming, first, the weak impact of the SNP on RapaCaspase9 suicide gene induction and, second, the use of the RapaCasp9-G suicide gene to improve the safety of our construct. In regard to the difference observed between lentiviral and retroviral constructs, we hypothesized that the different promoters in the constructs explain this variation, but this hypothesis needs to be validated with supplementary investigations.

To study this medication in conditions of a clinical trial, we have to be closer to clinical conditions and treatments given to future patients: for that, the next doses studied were calculated to be similar to taking a pill and we could confirm the efficiency of clinical doses for suicide gene induction. In addition, we showed, in vitro, that rapamycin (Rapamune®) pills are as efficient as rapamycin powder in treating RapaCasp9-G-expressing GMTCs. This supports that Rapamune® pills can be used in conditions of a clinical trial, even if additional studies need to be perform. Moreover, in the context of improving the safety of this therapy, the speed of suicide gene induction must be as fast as possible to quickly eliminate GMTCs. Even though induced cell death reached a plateau at 48 h after rapamycin exposure, most of the genetically modified cells were already eliminated after 24 h and no longer represented a danger to the patient. In comparison to other suicide gene systems for which induction is efficient between 4 and 6 days [36], the RapaCasp9-G suicide gene exhibited faster induction. In case of 24 h exposure to rapamycin is not enough to correctly induce suicide gene induction, we could plan to expose patients to a second consecutive dose, as it showed a better suicide gene induction than 24 h exposure (Supplementary Fig. S6). For the purpose of a clinical trial, the IL-1RAP CAR-T-cell production process must be GMP compliant. To ensure the efficiency of the RapaCasp9-G suicide gene in these conditions, the freezing and activation process must not have an impact on it: notably, the RapaCasp9-G suicide gene still showed the same functionality regardless of the activation process in HDs. Moreover, even without a rest day to restart GMTCs metabolism after thawing and in the presence or absence of target stimulation, high rapamycin-induced killing percentages confirmed the RapaCasp9-G suicide gene functionality.

More importantly, as therapy with IL-1RAP CAR-T cells is developed for AML patients, the functionality of the RapaCasp9-G suicide gene in RapaCasp9-G-expressing GMTCs produced from AML patients is an important milestone. Our experiments strongly confirmed this functionality and support the interest in suicide gene use in our CAR-T-cell therapy targeting the IL-1RAP protein in different activation processes. Unfortunately, the scarcity of samples from AML patients prevented us from performing further study.

Finally, an important characteristic of the CID is acceptable or limited side effects. As a side effect, rapamycin showed only transitory transgene expression changes, confirming the specific elimination of RapaCasp9-G-expressing GMTCs, and a slowdown in the proliferation of untransduced T cells, which could be turned into a potential advantage. Indeed, we can speculate that this effect could produce an observable impact on RapaCasp9-G-expressing GMTCs: in the case of suicide gene use, there could be a synergistic effect between the slowdown of T-cell metabolism, regardless of whether the T cells are genetically modified, and the specific elimination of RapaCasp9-G-expressing GMTCs. Moreover, rapamycin induced only transitory transgene expression changes and specifically eliminated GMTCs.

Taking into account the efficiency of rapamycin, its limited side effects, its “off-the-shelf” status and its specificity in eliminating target cells, this CID seems to be a great candidate as a suicide gene inducer. Furthermore, in order to better eliminate GMTCs in case of toxicities, a second suicide gene system could be added to enhance GMTCs elimination if necessary [37]. Moreover, the whole point of improving safety with a suicide gene is the association with a cell therapy in order to be as close as possible to the real conditions of use: this model thus perfectly meets the conditions for validated in vitro functionality.

## Data avaibility

Data are available at UMR1098 RIGHT research team, upon reasonable request to the first or last authors.

## Supplementary information

Supplemental material
