# Correction: RapaCaspase-9-based suicide gene applied to the safety of IL-1RAP CAR-T cells

**DOI:** 10.1038/s41434-024-00487-5

**Published:** 2024-12-20

**Authors:** Lucie Bouquet, Elodie Bôle-Richard, Walid Warda, Mathieu Neto Da Rocha, Rim Trad, Clémentine Nicod, Rafik Haderbache, Delphine Genin, Christophe Ferrand, Marina Deschamps

**Affiliations:** 1 Univ. Bourgogne Franche-Comté, INSERM, EFS BFC, UMR1098, RIGHT Interactions Greffon-Hôte-Tumeur/Ingénierie Cellulaire et Génique, F-25 000 Besançon, France; 2 CanCell Therapeutics, 25 000 Besançon, France

**Keywords:** Immunotherapy, Haematological cancer

**Correction to:** *Gene Therapy* (2023) **30**:706-713; 10.1038/s41434-023-00404-2; Article published online 12 May 2023

The figure 2 in the supplementary material has to be changed.

The initial figure, published as a supplementary data, was erroneously submitted from a draft figure version. We have therefore reformatted and corrected this figure. The legend remains unchanged. This does not affect the main message and conclusions of the paper.

The supplementary material has been corrected.

**Supplementary information** The online version contains supplementary material available at 10.1038/s41434-023-00404-2.

## Supplementary information

Supplementary information

