# Supplementary material for: RapaCaspase-9-based suicide gene applied to the safety of IL-1RAP CAR-T cells
Source: Gene Ther. 2023 May 12;30(9):706–13. doi: 10.1038/s41434-023-00404-2 (PMC10506905; doi:10.1038/s41434-023-00404-2)
Supplement: Supplementary file 1 — Supplemental material [file 41434_2023_404_MOESM1_ESM.pdf]

RAPACASPASE-9-BASED SUICIDE GENE APPLIED TO THE SAFETY OF IL-1RAP CAR-T CELLS

Lucie Bouquet, Walid Warda, Mathieu Neto Da Rocha, Rim Trad, Clémentine Nicod, Rafik Haderbache, Delphine Genin, Christophe Ferrand, Marina Deschamps

Supplemental Figures and Legends

- **Figure S1. Schematic representation of viral constructs.**
- **Figure S2. Generation of iCasp9-G and iCasp9-A retroviral plasmids.**
- **Figure S3. Generation of RapaCaspase9 suicide gene plasmids.**
- **Figure S4. Functional validation of RapaCasp9-G suicide gene, induced by rapamycin produced for research and Rapamune® pill.**
- **Figure S5 IL-1RAP stimulation validation by CD107a degranulation assay of RapaCasp9-G-expressing GMTCs**
- **Figure S6. Timing improvement for suicide gene induction in RapaCasp9-expressing GMTCs.**

Figure S1

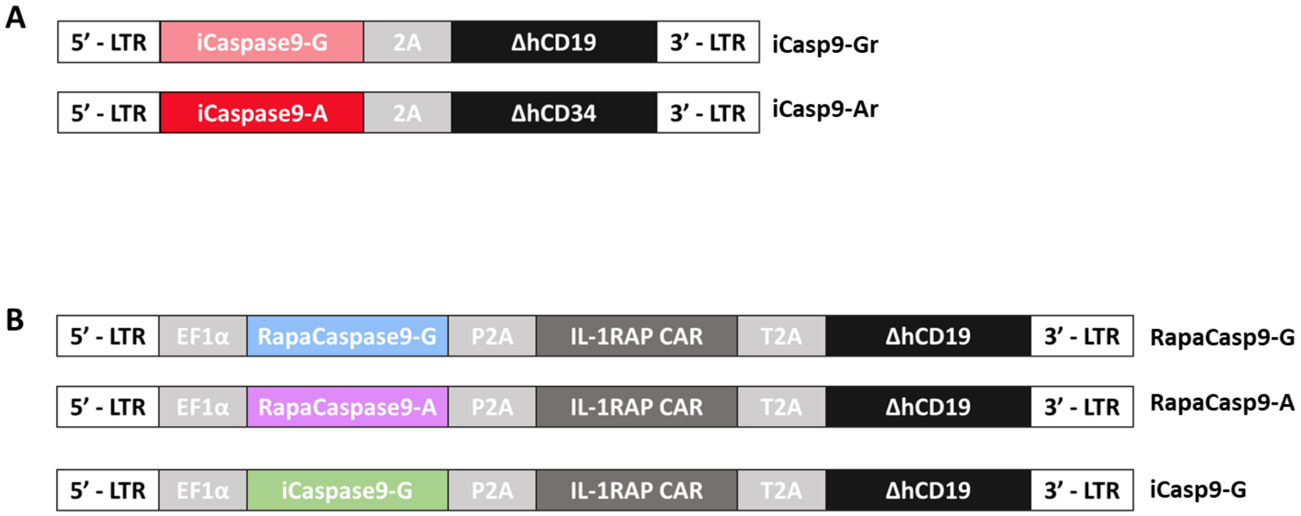

Figure S1. Schematic representation of viral constructs. A. Schematic representation of the retroviral iCaspase9 constructs, including the suicide gene and a ΔCD19. The different sequences are separated with 2a self-cleaving peptides. B. Schematic representation of the lentiviral RapaCaspase9 and iCaspase9 constructs, including an EF1α promoter, the suicide gene, an IL-1RAP chimeric antigen receptor and a ΔCD19. The different sequences are separated with 2a self-cleaving peptides

Figure S2

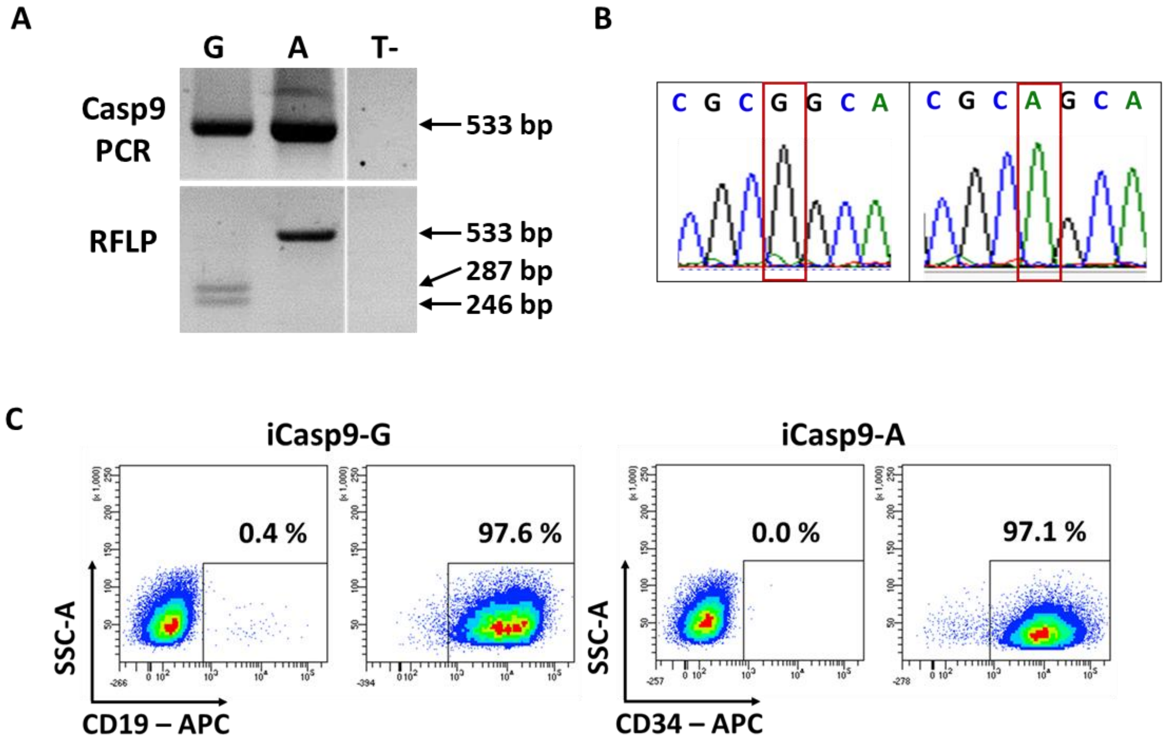

Figure S2. Generation of iCasp9-G and iCasp9-A retroviral plasmids. A. Restriction fragment length polymorphism of retroviral iCaspase9 plasmids targeting the site-directed mutagenesis site. PCR targeting the Casp9 suicide genes was performed, and the PCR products underwent enzymatic restriction with the *BstUI* enzyme and migration in an agarose gel. The negative control (T-) contained water instead of DNA. B. Validation of the site-directed mutagenesis by Sanger sequencing of the iCaspase9 suicide genes. C. Transfection efficiency of PG13-iCasp9-G and PG13-iCasp9-A after sorting with CD19 or CD34 labeling.

Figure S3

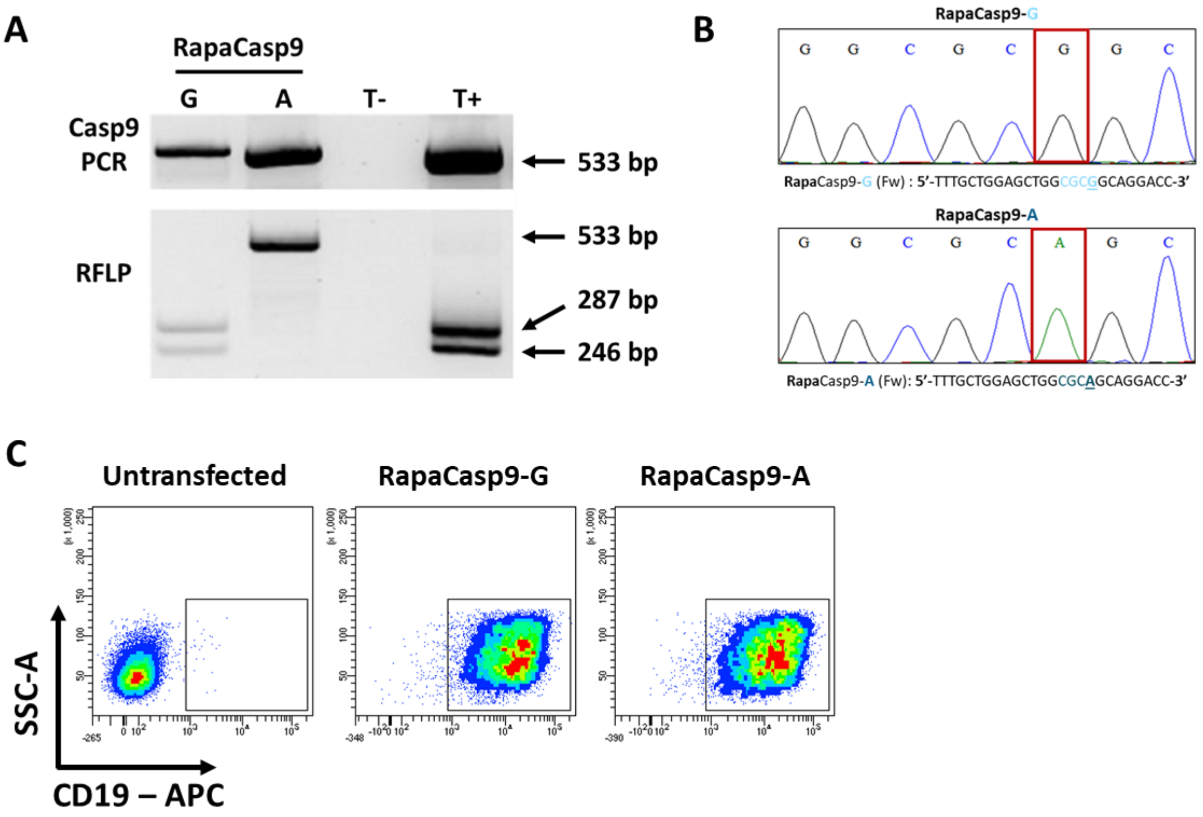

Figure S3. Generation of RapaCaspase9 suicide gene plasmids. A. Restriction fragment length polymorphism of lentiviral RapaCaspase9 plasmids targeting the site-directed mutagenesis site. PCR targeting the Casp9 suicide genes was performed, and the PCR products underwent enzymatic restriction with the *BstUI* enzyme and migration in an agarose gel. The negative control (T-) contained water instead of DNA, and the positive control (T+) contained a validated plasmid with a G nucleotide. B. Validation of the site-directed mutagenesis by Sanger sequencing of the Casp9 suicide genes. C. Representative dot plots of the transfection efficiency for HEK-293T cells transfected with RapaCasp9-G and RapaCasp9-A lentiviral plasmids.

Figure S4

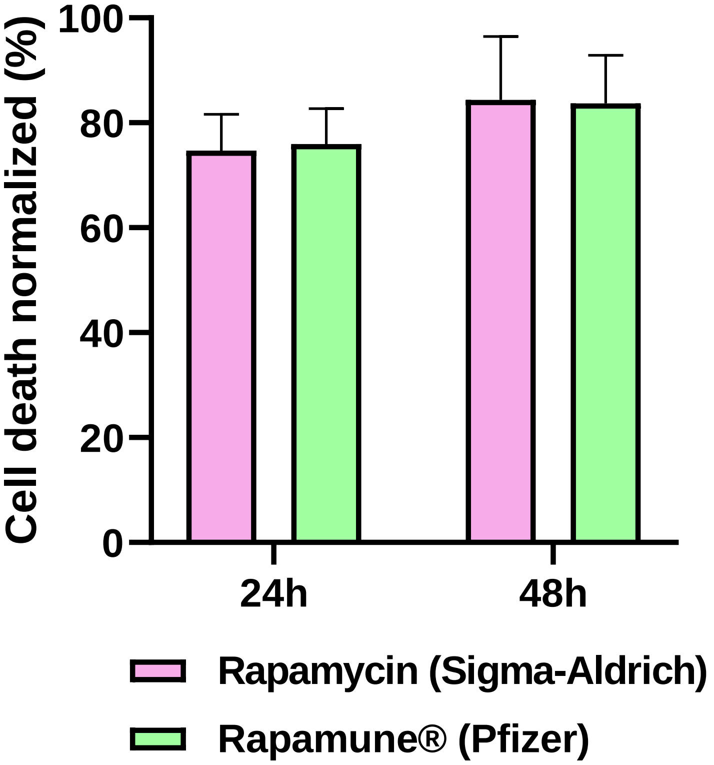

Figure S4. Functional validation of RapaCasp9-G suicide gene, induced by rapamycin produced for research and Rapamune® pill. Cell death percentages of RapaCasp9-G-expressing GMTCs during 24 and 48 hours after rapamycin exposure. Cells were exposed to 109 nM rapamycin from Sigma-Aldrich (produced for research) or from Rapamune® pill (commercialized rapamycin), and normalized to control cells (DMSO treated). Cell death was evaluated by Annexin-V/7-AAD labeling and gating on $CD3^+/CD19^+$ cells by flow cytometry. Mean ± SD of three independent experiments.

Figure S5

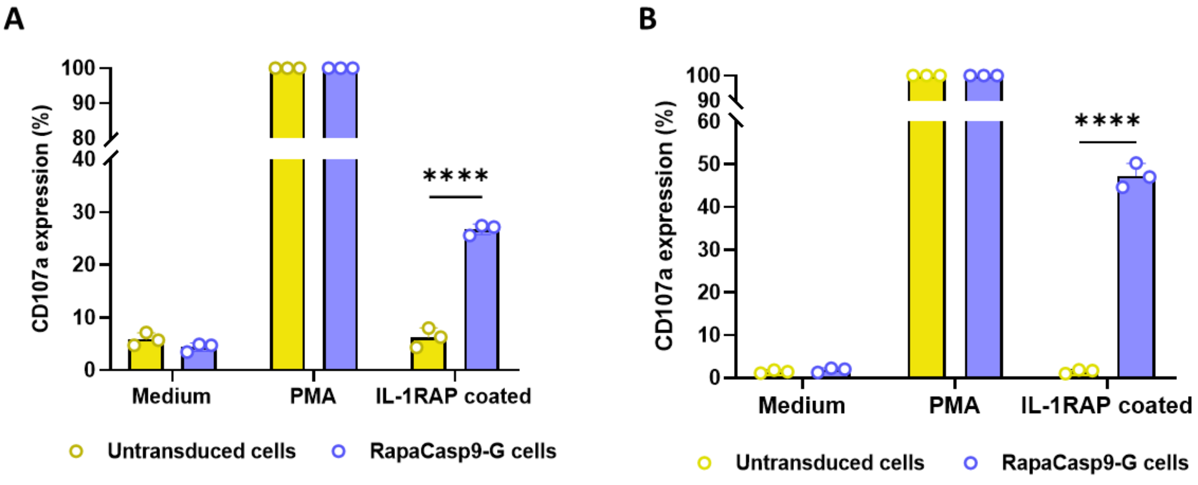

Figure S5. IL-1RAP stimulation validation by CD107a degranulation assay of RapaCasp9-G-expressing GMTCs. Analysis of CD107a degranulation assay of RapaCasp9-G-expressing GMTCs cells after stimulation with IL-1RAP bounded protein during 24 hours **A.** Percentages of total $CD3^+/CD19^+/CD8^+/CD107a^+$ in untransduced or RapaCasp9-expressing GMTCs from AML patients. Mean ± SD of three independent experiments. **** P<0.0001 with two-way Anova**. B/** Percentages of total $CD3^+/CD19^+/CD8^+/CD107a^+$ in untransduced or RapaCasp9-expressing GMTCs from HDs after thawing. Mean ± SD of three independent experiments. **** P<0.0001 with two-way Anova**.**

Figure S6

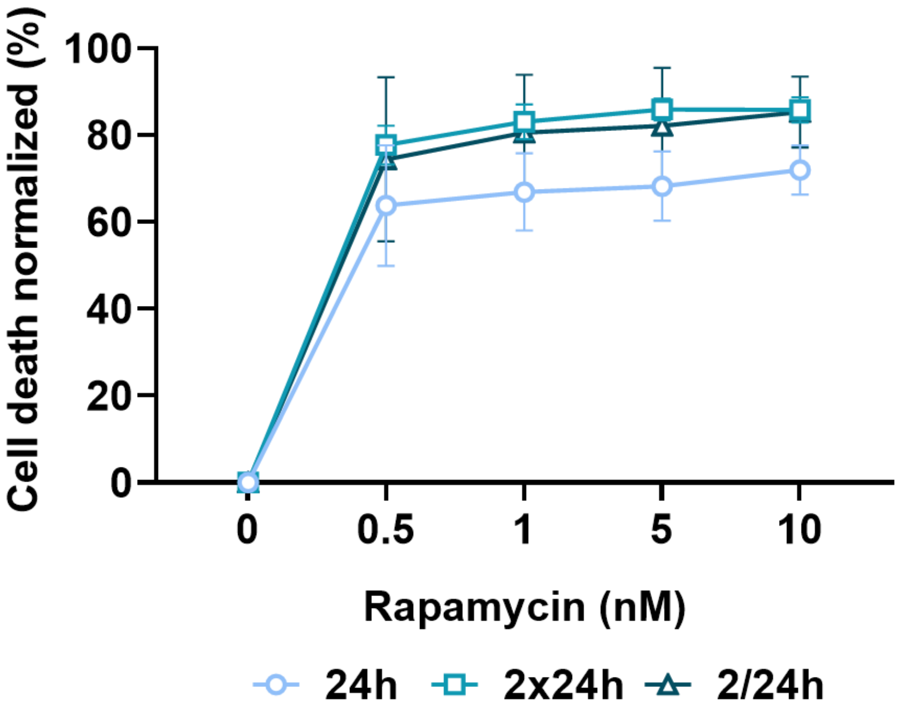

Figure S6. Timing improvement for suicide gene induction in RapaCasp9-expressing GMTCs. Killing curve after exposure to rapamycin at doses ranging from 0.5 nM to 10 nM. RapaCasp9-G-expressing GMTCs were exposed to rapamycin during 24 hours (24h), to two successive doses of rapamycin during 24 hours with cells washing between each dose (2x24h), and two successive half-doses of rapamycin added 24 hours apart (2/24h). Mean ± SD of three independent experiments.
